# T Cell Protein Tyrosine Phosphatase in Osteoimmunology

**DOI:** 10.3389/fimmu.2021.620333

**Published:** 2021-02-22

**Authors:** Ya-nan Wang, Shiyue Liu, Tingting Jia, Yao Feng, Wenjing Zhang, Xin Xu, Dongjiao Zhang

**Affiliations:** ^1^ Department of Implantology, School and Hospital of Stomatology, Cheeloo College of Medicine, Shandong University, Jinan, China; ^2^ Shandong Key Laboratory of Oral Tissue Regeneration, Jinan, China; ^3^ Shandong Engineering Laboratory for Dental Materials and Oral Tissue Regeneration, Jinan, China; ^4^ Department of Periodontology, School and Hospital of Stomatology, Cheeloo College of Medicine, Shandong University, Jinan, China

**Keywords:** T cell protein tyrosine phosphatase (TCPTP), Protein tyrosine phosphatase non-receptor 2 (PTPN2), osteoimmunology, macrophages, T cell, B cell

## Abstract

Osteoimmunology highlights the two-way communication between bone and immune cells. T cell protein tyrosine phosphatase (TCPTP), also known as protein-tyrosine phosphatase non-receptor 2 (PTPN2), is an intracellular protein tyrosine phosphatase (PTP) essential in regulating immune responses and bone metabolism *via* dephosphorylating target proteins. *Tcptp* knockout in systemic or specific immune cells can seriously damage the immune function, resulting in bone metabolism disorders. This review provided fresh insights into the potential role of TCPTP in osteoimmunology. Overall, the regulation of osteoimmunology by TCPTP is extremely complicated. TCPTP negatively regulates macrophages activation and inflammatory factors secretion to inhibit bone resorption. TCPTP regulates T lymphocytes differentiation and T lymphocytes-related cytokines signaling to maintain bone homeostasis. TCPTP is also expected to regulate bone metabolism by targeting B lymphocytes under certain time and conditions. This review offers a comprehensive update on the roles of TCPTP in osteoimmunology, which can be a promising target for the prevention and treatment of inflammatory bone loss.

## Introduction

Inflammatory bone diseases characterized by severe bone loss, such as osteoarthritis, rheumatoid arthritis, and periodontitis, are a manifestation of imbalance between the skeletal and immune systems (1–3). Their interactions, known as osteoimmunology, were firstly proposed by Choi and Aaron in 2000, which highlights the two-way communication between bone and immune cells (4). With a comprehensive and profound acknowledgment of osteoimmunology, targeting regulatory proteins involved in osteoimmune responses can be a feasible means against inflammatory bone diseases. T-cell protein tyrosine phosphatase (TCPTP), one of the protein tyrosine phosphatases (PTPs) family, was identified by Cool et al. using T-cell-based cDNA library screening (5). There is growing evidence that TCPTP is a critical regulator in immune responses and bone metabolism. Nevertheless, the potential effect of TCPTP in the field of osteoimmunology is less explored. As a result, we intended to offer a comprehensive update on the known and potential roles of TCPTP in osteoimmunology in this review. Our study will lay a theoretical foundation for further basic researches of TCPTP in the field of osteoimmunology and provide references for treatment strategies of inflammatory bone diseases.

## Osteoimmunology

**Figure 1:**
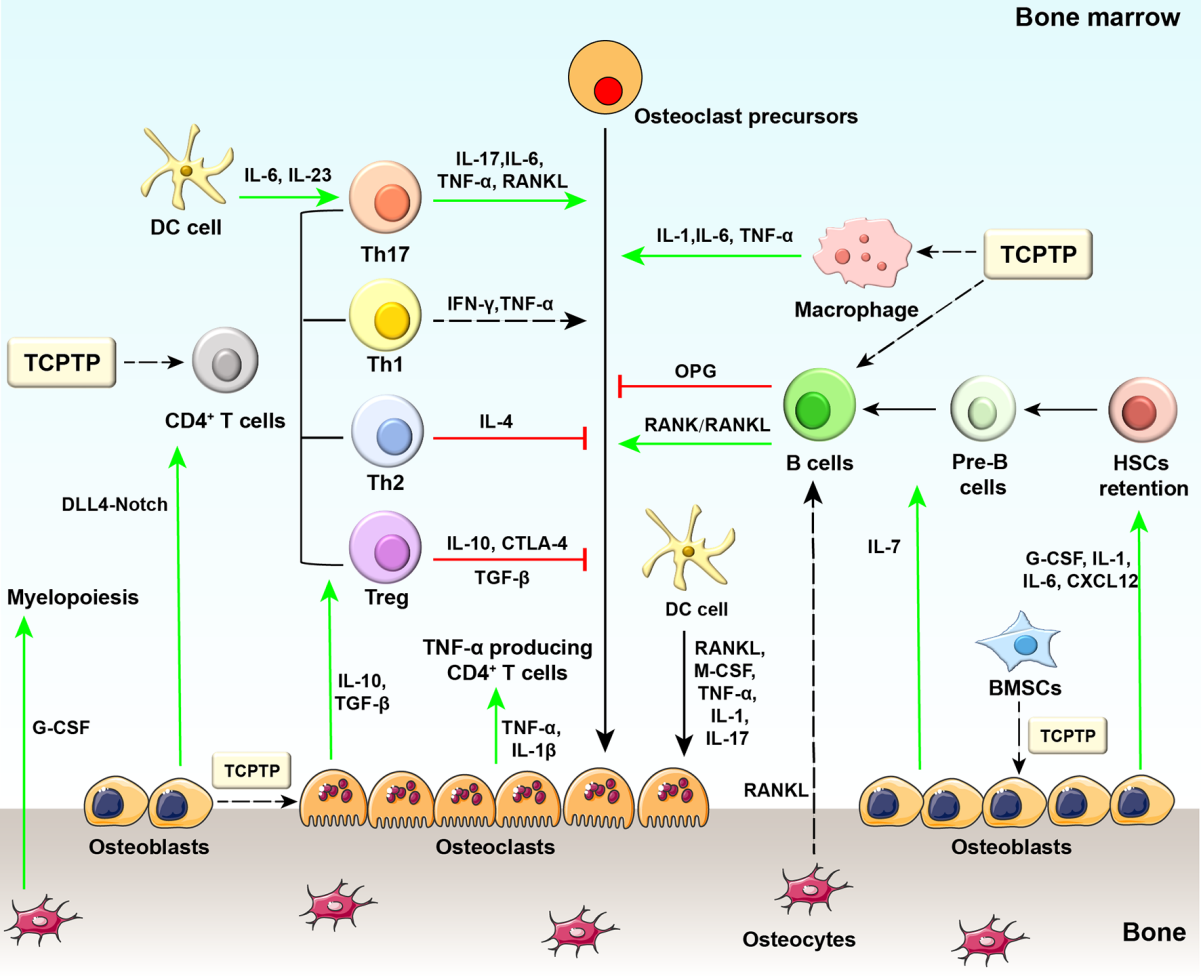
Diagram of osteoimmunology and potential regulatory sites of TCPTP. Regarding the regulation of bone-related cells by immune cells, some CD4^+^ T cell subsets can produce osteoclastogenic cytokines (e.g., TNF-α from Th1 cells and IL−17 from Th17 cells), while other subsets secrete anti-osteoclastogenic cytokines (e.g., IL−4 from Th2 cells, IL−10 and CTLA4 from Treg cells). IFN-γ released by Th1 cells may exert both pro- and anti-osteoclastogenic effects as reported by previous studies. B cells physiologically inhibit osteoclastogenesis but stimulate osteoclastogenesis through activating the RANK/RANKL axis in the pathological state. Different stages of dendritic cells exhibit distinct properties in immune responses: immature dendritic cells differentiate into osteoclasts in response to M-CSF, RANKL, TNF-α, IL-1, and IL-17, while mature ones drive the activation and expansion of Th17 cells. Macrophages are essential to bone loss with the involvement of TNF-α, IL-1, and IL-6 released by themselves. On the other hand, bone-related cells also provide feedback to immune cells. Osteoblasts secrete G-CSF, IL−1, IL−6, IL−7, and CXCL12, which are required for HSCs maintenance. In addition, osteoblasts secrete IL-7 to support B lymphopoiesis and regulate DLL4-Notch signaling pathway to support T lymphopoiesis. Osteocytes are supposed to mobilize HSCs and are involved in myelopoiesis. Osteocyte-derived RANKL participates in estrogen deficiency-induced bone loss by indirect regulation of B cell development. Osteoclasts are also involved in antigen presentation and T cell activation. Osteoclasts secrete tolerogenic cytokines (e.g., IL-10, TGF-β) and activate regulatory T cells in physiological conditions, while in pathological conditions, osteoclasts secrete inflammatory cytokines (e.g., TNFα, IL-1β) and activate TNF-α producing CD4^+^ T cells. Generally, TCPTP regulates bone metabolism mainly by changing the biofunction of macrophages, T cells, and B cells. TCPTP, T cell protein tyrosine phosphatase; DC cell, dendritic cell; IL-6, interleukin-6; IL-23, interleukin-23; IL-17, interleukin-17; TNF-α, tumor necrosis factor-α; RANK, receptor activator of nuclear factor−κB; RANKL, receptor activator of nuclear factor−κB ligand; IL-1, interleukin-1; IL-4, interleukin-4; IL-10, interleukin-10; CTLA4, cytotoxic T lymphocyte protein 4; OPG, osteoprotegerin; Th17, T helper 17 cells; Th1, T helper 1 cells; Th2, T helper 2 cells; Treg, regulatory T cells; G-CSF, granulocyte colony-stimulating factor; M-CSF, macrophage colony-stimulating factor; TGF-β, transforming growth factor β; DLL4, Delta-like protein 4; IL-1β, interleukin-1β; IL-7, interleukin-7; CXCL12, CXC-motif chemokine 12; HSCs, hematopoietic stem cells.

During osteoimmune responses, different immune cells and bone cells interact reciprocally to maintain the homeostasis between the immune and skeletal systems (**Figure 1**). The two-way communication may influence either immune or bone cells *via* cytokine activities in the immune-bone interface. The proposal of screening new targets interfering with osteoimmunology may be feasible for identifying critical targets suppressing immune hyperactivity in inflammatory bone loss diseases.

### Influence of Immune Cells on Bone Cells

Immune cells, such as T lymphocytes (Th1, Th2, Treg, and Th17 cells), B lymphocytes, dendritic cells, and macrophages, actively regulate the homeostasis of bone metabolism. Th1 cells secrete interferon-γ (IFN-γ) that has been found to exert controversial effects in bone metabolism (6, 7). Sato et al. demonstrated that Th1 cells generated amounts of IFN-γ and mediated osteoclastogenesis inhibition *in vitro* (8). However, another study reported that IFN-γ could promote osteoclast maturation in the late period of osteoclastogenesis (9). Th1 cells are found to induce orthodontic tooth movement and bone resorption indirectly by upregulating the tumor necrosis factor-alpha (TNF-α) secretion and promoting osteoclastogenesis (10, 11). Th2 cells secrete interleukin-4 (IL-4), interleukin-5 (IL-5), and interleukin-13 (IL-13) leading to the osteoclastogenesis inhibition in a signal transducer and activator of transcription 6 (STAT6)-dependent pathway (12). Cytotoxic T-lymphocyte antigen 4 (CTLA4) secreted by Treg cells can promote apoptosis of osteoclasts *via* binding to CD80/CD86 on osteoclast precursors (13). Besides, Treg cells not only inhibit osteoclastogenesis directly *via* suppressing receptor activator of nuclear factor-κB ligand (RANKL) generation (14) but also suppress osteoclast differentiation and bone resorption by secreting interleukin-10 (IL-10) and transforming growth factor-β (TGF-β) (15). Th17 cells are one of the osteoclastogenic subsets of T cells that participate in various inflammatory diseases, such as rheumatoid arthritis, osteoporosis, inflammatory bowel disease, and periodontal disease (16–18). In the process of osteoclastogenesis and bone loss, higher amounts of osteoclastogenic cytokines, including interleukin-17 (IL-17), interleukin-6 (IL-6), interleukin-1 (IL-1), and TNF-α, are released from Th17 cells (19, 20). Among these cytokines, IL-17 stimulates the synthesis of cyclooxygenase-2 dependent prostaglandin E2 and the gene transcription of osteoclast differentiation factor (ODF) in osteoblasts to induce osteoclastogenesis (21). B cells inhibit osteoclastogenesis *via* secreting osteoprotegerin in the physiological state but stimulate osteoclastogenesis through activating the receptor activator of nuclear factor−κB (RANK)/RANKL axis in the pathological state (22, 23). Human immature dendritic cells differentiate into osteoclasts in response to macrophage colony-stimulating factor (M-CSF), RANKL, TNF-α, IL-1, and IL-17 (24–26), while mature dendritic cells can drive the activation of Th17 cells that produce IL-17, thereby enhancing osteoclastogenesis (27). Macrophages are reported to secrete different proinflammatory cytokines (e.g., TNF-α, IL-1, and IL-6) to enhance bone loss (28).

### Influence of Bone Cells on Immune Cells

Bone-related cells (e.g., osteoblasts, osteocytes, osteoclasts) could also regulate the immune system. In this process, numerous cytokines (e.g., granulocyte colony-stimulating factor [G−CSF], IL−1, IL−6, IL−7, and CXC-motif chemokine 12 [CXCL12]) are secreted from osteoblasts for hematopoietic stem cell (HSC) maintenance, lymphoid progenitor cell maintenance, as well as the balance of T cell or B cell generation (29–31). Zhu et al. reported that osteoblasts support all stages of B lymphopoiesis *via* locally secreting interleukin-7 (IL-7) and stromal cell derived factor-1 (SDF-1) (32, 33). Osteoblasts-specific knockout of osteocalcin results in a marked reduction in mature T cells through disrupting the delta-like protein 4-notch signaling (34). Osteocytes are supposed to mobilize hematopoietic stem cells (HSCs) and might also be involved in myelopoiesis. In mice with targeted ablation of osteocytes, the mobilization of HSCs was suppressed in bone marrow (35). Besides, deficiency of the G−protein subunit GSα in osteocytes results in increased G−CSF production and dramatic expansion of myeloid lineage cells (36). RANKL generated by osteocytes participates in estrogen deficiency-induced bone loss by regulating B cell development indirectly (37). Several studies reported that osteoclasts could promote the mobilization of hematopoietic progenitor cells (38), while others revealed that they were dispensable for HSC maintenance and mobilization (39). Osteoclasts are also involved in antigen presentation and T cell activation. In the physiological conditions, osteoclasts secrete tolerogenic cytokines (such as IL-10 and TGF-β) and activate CD4^+^ and CD8^+^ regulatory T cells, while in the pathological conditions, osteoclasts activate TNF-α producing CD4^+^ T cells *via* unleashing myriads of inflammatory cytokines (e.g., TNF-α and IL-1) (26, 40, 41).

## TCPTP

### Overview of TCPTP

TCPTP is a tyrosine-specific phosphatase that is firstly identified by Cool et al. (5). There are two splice variants of TCPTP: TC45 (45 kDa) which is located in nuclear and TC48 (48 kDa) which is located in the endoplasmic reticulum (42). TC45 is a widely expressed form in various species, including humans and mice, and TC48 is human-specific. In most species, TC45 shuttles between the nucleus and cytoplasm in response to cytokine stimulation (43).

TCPTP regulates diverse signaling pathways related to glucose metabolism, inflammation control, cancer progress, and other biological processes *via* dephosphorylation of distinct substrates (44–50). Experiments *in vitro* and *in vivo* have confirmed that TCPTP could regulate several cytokine signaling pathways by inhibiting Janus activated kinase (JAK)/signal transducer and activator of transcription (STAT) predominantly (51–54). The direct substrates have been recognized as JAK1, JAK3, STAT1, STAT3, and STAT5 (55–59). Some members of the tyrosine kinase receptor (RTK) family, comprising insulin receptors (IRs) (60, 61), epidermal growth factor receptors (EGFRs) (62, 63), vascular endothelial growth factor receptors (VEGFRs) (64), platelet-derived growth factor receptors (PDGFRs) (65, 66) and colony-stimulating factor-1 receptors (CSF-1Rs) are also the specific dephosphorylating substrates of TCPTP (67).

### TCPTP and Immunomodulation

Growing evidence has indicated that TCPTP is a key player in regulating innate and acquired immune responses. GWA studies found single nucleotide polymorphisms (SNPs) of TCPTP are associated with the onset of several inflammatory diseases and autoimmunology disorders, such as inflammatory bowel disease (68, 69), ocular Behcet’s disease (70), rheumatoid arthritis (53), and juvenile inflammatory arthritis (71). *Tcptp* knockout in the systemic or specific cells can seriously jeopardize immune reactions.

Mice null for *Tcptp* (*Tcptp* ^−/−^) showed a smaller body size, decreased mobility, severe anemia, and diarrhea followed by death at three to 5 weeks of age (72). From the perspective of histology, *Tcptp* ^−/−^ mice showed mononuclear cell infiltration in the salivary gland and gastric mucosa at 1 week of age (72). Dramatic increases in TNF-α and inducible nitric oxide synthase (iNOS) were also detected at 3 weeks of age in *Tcptp* ^−/−^ mice (73).

Loss of PTPN2 in T cells (TCPTP-CD4Cre) not only led to increased intestinal inflammation risk but also resulted in T-lymphocyte infiltration in the liver, kidney, and skin (74, 75). This may be caused by the enhanced induction of Th1 cells, Th17 cells, and effector and memory CD8^+^ T cells, but the impaired induction of Tregs after T cell-specific knockout of *Tcptp*. Myeloid cell-specific loss of TCPTP (TCPTP-LysMCre) also enhanced susceptibility to colitis and serum IL-1β levels in mice (76). Spalinger et al. reported that TCPTP loss in macrophages compromises epithelial cell-macrophage interactions and reduces epithelial barrier integrity (77). Furthermore, TCPTP knockdown in THP-1 cells elevated the IFN-γ-induced secretion of the proinflammatory cytokines IL-6 (78). TCPTP silencing in rheumatoid arthritis synovial fibroblasts could also increase IL-6 production (53).

### TCPTP and Hematopoiesis

Although ubiquitously expressed, TCPTP is pronouncedly expressed in hematopoietic tissues and plays a significant role in the development of hematopoietic lineages (5).

#### TCPTP and Hematopoiesis: Stem Cells

Hematopoietic stem cells (HSCs) are one of the adult stem cells and can differentiate into various mature blood cells (79). Compared with the control group, *Tcptp* knockout resulted in a nine-fold increase of the HSC number in the bone marrow (80). Lymphoid and myeloid precursors were also more abundant in *Tcptp* ^−/−^ mice compared with the wide type controls (45). Bourdeau et al. also demonstrated that this effect could be reproduced by TCPTP inhibiting agents and interleukin-18 (IL-18) signaling pathway involved in this process (80). The above results implicated that TCPTP plays an important role in the regulation of HSC proliferation.

#### TCPTP and Hematopoiesis: Myeloid Cells

*Tcptp* ^−/−^ mice exhibited increased splenic macrophage populations and yielded four times of the macrophage colony-forming unit (CFU-M) number (67). TCPTP is also involved in myeloid progenitor development. *Tcptp* ^−/−^ mice had 4 times of the granulocyte/macrophage precursors (GMPs) compared with wildtype mice and CSF-1/CSF-1R signaling may involve in this process (67).

*Tcptp* ^−/−^ mice were reported to suffer from severe anemia which could contribute to their early lethality. You-Ten et al. reported a failure to initiate hematopoietic function in the bone marrow of *Tcptp* ^−/−^ animals after 2–3 weeks (72). What’s more, the deficiency of bone marrow stromal cell numbers, the impairment of remaining stromal cells, and the inadequate cytokines production by the bone marrow microenvironment could be the possible explanations for the defective hematopoiesis in *Tcptp* ^−/−^ mice (72).

#### TCPTP and Hematopoiesis: Lymphocytes

*Tcptp* ^−/−^ mice exhibited specific defects of B cell lymphopoiesis in the bone marrow, however, T cell development in the thymus was not significantly affected (72). The defects of B cell lymphopoiesis were characterized by fewer pre-B cell colonies and impaired transition to the immature B-cell stage (81). Bourdeau et al. have found that bone marrow stromal cells from *Tcptp* ^−/−^ mice could secrete higher levels of IFN-γ resulting in a 2-fold reduction in the mitotic index on IL-7 stimulation of *Tcptp* ^−/−^ pre-B cells (81).

### TCPTP and Bone Metabolism

Recent studies using gene knockout mice have emphasized the importance of TCPTP in bone metabolism. *Tcptp* ^−/−^ BALB/c mice showed significantly reduced femoral length and width, as reflected by the large volume of unabsorbed cartilage at the epiphysis by 14 days of age (82); however the latter was no longer evident at 21 days of age (82). Besides, *Tcptp* ^−/−^ mice have a higher incidence of synovitis in the knee joint (83). Loh et al. found that the runted body of neuronal cell-specific *Tcptp* knockout mice is associated with decreased circulating growth hormone (84). However, the precise mechanisms that underlie the bone development in *Tcptp* ^−/−^ mice still need further investigation.

Insulin signaling in osteoblast inhibits the expression of osteoprotegerin (OPG), an osteoclastogenesis inhibitory factor (85). TCPTP expressed in osteoblasts regulates insulin receptor phosphorylation, thus activating insulin signaling (86). A classical coculture assay revealed that osteoblasts lacking TCPTP induced more tartrate-resistant acid phosphatase (TRAP) positive osteoclasts than wild-type osteoblasts did (86). Accordingly, osteoclast activity was increased in osteoblasts-specific *Tcptp* ^−/−^ mice which was proved by increased serum levels of CTx, a marker of bone resorption (86).

## TCPTP in Osteoimmunology

As shown in **Figure 1**, TCPTP could influence bone metabolism by regulating the biofunction of macrophages, T cells, and B cells. Besides, TCPTP in osteoblasts and bone marrow stem cells also regulates bone metabolism. The potential roles of TCPTP in osteoimmunology are discussed below.

### TCPTP and Macrophages in Osteoimmunology

**Figure 2:**
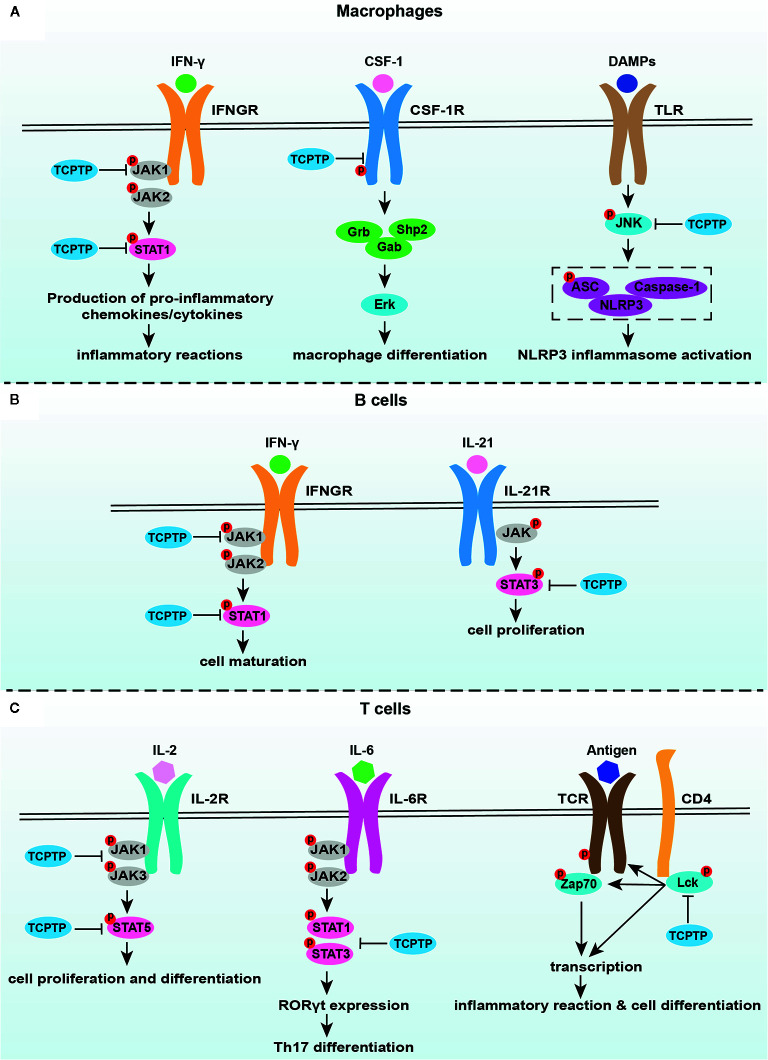
TCPTP-related signaling pathways in macrophages, B cells, and T cells. **(A)** TCPTP downregulates the IFN-γ signaling, the CSF-1/CSF-1R signaling, and inflammasome activation in macrophages. **(B)** TCPTP drives B cell maturation *via* suppressing the IFN-γ signaling and prohibits B cell proliferation by downregulating the IL-21 signaling. **(C)** TCPTP inhibits IL-6-driven pathogenic loss of Foxp3 after Tregs have acquired RORγt expression through dephosphorylation of STAT3. TCPTP negatively regulates the IL-2 receptor signaling by JAK1, JAK3, and STAT5 dephosphorylation, which is an important way related to Treg differentiation. TCPTP inhibits TCR activation and hyperphosphorylation of Lck to inhibit inflammatory reactions and cell differentiation. TCPTP, T cell protein tyrosine phosphatase; JAK1, Janus activated kinase 1; JAK2, Janus activated kinase 2; JAK3, Janus activated kinase 3; STAT1, signal transducer and activator of transcription1; STAT3, signal transducer and activator of transcription3; STAT5, signal transducer and activator of transcription5; IFN-γ, interferon-γ; IFNGR, interferon-γ receptor; CSF-1, colony-stimulating factor 1; CSF-1R, colony-stimulating factor 1 receptor; DAMPs, damage-associated molecular pattern molecules; TLR, toll-like receptor; JNK, c-Jun N-terminal kinase; IL-21, interleukin-21; IL-21R, interleukin-21R; IL-6, interleukin-6; IL-6R, interleukin-6R; TCR, T cell receptor.

Macrophages, a significant component of the non-specific immunity, are crucial regulators in bone metabolism and can be regulated by TCPTP (77, 87, 88). The potential influences of TCPTP on osteoimmunology through macrophages can be expounded from four aspects. Firstly, TCPTP negatively regulates macrophages development (45). *Tcptp* ^−/−^ animals exhibited significantly impaired bone marrow microenvironment (including impairment of erythropoiesis, the decline of pre-B and mature B cells, and reduced stromal cells) and increased macrophage numbers (89). Secondly, TCPTP participates in the process of macrophage polarization. M1 and M2 macrophages, the two major phenotypes of macrophages, are pro-inflammatory and anti-inflammatory respectively. A previous study reported that the increased M1/M2 ratio finally promoted osteoclastogenesis (90), and TCPTP could reverse diabetes-mediated high M1/M2 polarization in mice (91). This can be attributed to the fact that macrophages from *Tcptp* ^−/−^ mice are inherently hypersensitive to lipopolysaccharide and IFN-γ stimulation, which are two main cytokines that induce M1 differentiation (73, 92). Thirdly, the colony-stimulating factor 1 (CSF-1)/colony-stimulating factor 1 receptor (CSF-1R) signaling has been proven to be downregulated by TCPTP in macrophages. After CSF-1 stimulation, tyrosine phosphorylation of the CSF-1R markedly increased, and the activation of extracellular regulated protein kinases (ERK) was enhanced in *Tcptp* ^−/−^ macrophages (67). Zhang et al. ascertained that TCPTP inhibited alveolar bone resorption in diabetic periodontitis *via* dephosphorylating CSF-1R at the Y807 site, thereby prohibiting osteoclast differentiation (93). Fourthly, the dephosphorylating of c-Jun N-terminal kinase (JNK) in macrophages is another means how TCPTP protects against inflammatory response and bone loss caused by inflammasome-mediated interleukin-1β secretion (76). These studies suggest that TCPTP may be a potential treatment target against inflammatory bone loss induced by macrophage-related disorders, for example, periodontitis and synovitis (**Figure 2A**).

### TCPTP and T Cells in Osteoimmunology

Undoubtedly, T cells play a critical role in bone homeostasis, wherein the effects of TCPTP on T cell function cannot be neglected (**Figure 2C**). TCPTP negatively regulates T cell activation (94, 95). *Tcptp* ^−/−^ T cells show enhanced cell activity *via* the reduction of T cell receptor (TCR) threshold and hyperphosphorylation of the activated tyrosine residue of Lck (75). Furthermore, TCPTP regulates T cell differentiation (91, 96, 97). TCPTP regulated the activation and differentiation of T cells in colonic inflammation (96). Wiede et al. showed that TCPTP attenuates the activity of the STAT5 signaling to regulate αβ TCR versus γδ TCR T cell development (97). Li et al. reported that TCPTP overexpression reversed the high Th1/Treg and Th17/Treg ratios in epididymal white adipose tissue of diabetic mice (91). Spalinger et al. showed that *Tcptp* ^−/−^ CD4^+^ T cell reinfusion led to a nearly 3-fold increase in the frequency of Th1 cells, a 2-fold increase in the frequency of Th17 cells, and by contrast, a 3-fold decrease in the frequency of Tregs in colitis animal models (74). In addition, the inhibition of the interleukin-2 (IL-2)/IL-2 receptor pathway mediated by JAK1, JAK3, and STAT5 dephosphorylation was found to be the mechanism of how TCPTP drives Treg differentiation possibly (98). As illustrated above, various cytokines (e.g., IFN-γ and IL-6 from Th1 cells) released from stimulated T cells bridge the two-way communications in osteoimmune responses. These two cytokines, which are closely associated with osteoimmunology, can be regulated by TCPTP. Therefore, the interactions between the two cytokines and TCPTP in bone metabolism were discussed in detail below.

#### TCPTP and IFN-γ

IFN-γ is a classical cytokine secreted from Th1 cells. On the one hand, IFN-γ promotes osteoclastogenesis *via* promoting the fusion of mononucleated pre-osteoclasts in the late period of osteoclastogenesis directly and stimulating the secretion of osteoclastic factors (such as RANKL and TNF-α)indirectly (9, 99, 100). By contrast, IFN-γ can also intensively suppress osteoclastogenesis by degrading tumor necrosis factor receptor-associated factor 6 and inhibiting the RANK/RANKL signaling pathway (6). TCPTP downregulates the IFN-γ signaling through JAK1, STAT1, and STAT3 dephosphorylation (52, 55, 59, 92). So, a rational thread of targeting TCPTP to suppress IFN-γ activity is expected to regulate osteoclastogenesis, and this hypothesis still needs more validations.

#### TCPTP and IL-6

IL-6 is an inflammatory cytokine that exerts pathological effects on inflammatory bone loss (101, 102), directly supporting osteoclast formation, stimulating osteoclast differentiation, and accelerating bone resorption (103, 104). IL-6 is associated with Th17 cell differentiation (105), and abnormalities in the IL-6 signaling can disturb the Th17/Treg balance and influence bone homeostasis indirectly (106). Concerning the regulation of IL-6 by TCPTP, TCPTP curbs both IL-6 secretion and signaling. Aradi et al. confirmed that TCPTP silencing significantly increased IL-6 secretion from synovial fibroblasts in rheumatoid arthritis animals (53). TCPTP also dephosphorylate STAT3 at the Y705 site, thereby suppressing IL-6 signaling (107, 108). Besides, TCPTP inhibits the IL-6-driven pathogenic loss of Foxp3 after Tregs have acquired RORγt expression through dephosphorylation of STAT3 (109). Therefore, TCPTP can be expected to inhibit bone resorption through inhibiting IL-6 generation and IL-6 signaling directly or reversing IL-6-induced Th17/Treg imbalance indirectly.

### TCPTP and B Cells in Osteoimmunology

B lymphocytes play an extremely vital role in both immune responses and bone metabolism, but the detailed relationship between B lymphocytes and osteoclastogenesis remains some points for debate. Physiologically, B cells produce osteoprotegerin to inhibit osteoclastogenesis but stimulate osteoclastogenesis *via* the RANK/RANKL axis in the pathological state (22, 23, 110). Recently, increasing evidence tended to support that B cell development and proliferation were crucially affected by TCPTP (**Figure 2B**). An impaired transition from pre-B to immature B cell was found in *Tcptp* ^−/−^ mice (45, 89), which might be associated with the abnormally increased release of IFN-γ and enhanced STAT1 phosphorylation in the pre-B cell compartment (81). With interleukin-21-induced hyperactivity of the STAT-3 signaling in *Tcptp* ^−/−^ mice, B cell proliferation was simultaneously boosted (111). These results ascertain TCPTP as an important regulator of B cells in bone homeostasis. However, concerning the complexity of the two-way effect between B lymphocytes and bone homeostasis or between TCPTP and B lymphocytes, the specific effect and the mechanisms behind each regulation should be confirmed in further explorations.

### TCPTP and Bone-Related Cells in Osteoimmunology

TCPTP that is expressed in osteoblasts and bone marrow stem cells participates in the regulation of bone metabolism (**Figure 1**). Zee et al. demonstrated that TCPTP deficiency in osteoblasts enhanced the activity of osteoclasts by activating insulin signaling and inhibiting the expression of OPG *in vitro (* 86). Our previous study found that TCPTP improved the osteogenic differentiation ability *via* ERK dephosphorylation in rat bone marrow stem cells in high glucose conditions (112). However, to our knowledge, no specific studies have reported the impacts of TCPTP in bone-related cells on immune cells which may be a novel aspect in the future, and the underlying mechanism also needs further exploration.

## Therapeutic Aspects From “TCPTP and Osteoimmunology” Perspective

TCPTP exerts an anti-inflammatory role in innate and adaptive immunity (113). As described in Section *TCPTP and Immunomodulation*, *Tcptp* knockout in systemic or specific immune cells brings about serious immune disorders. TCPTP deficiency also leads to subchondral bone loss and spontaneous synovitis mediated by excessive inflammatory cytokines (83). Loss-of-function variants of TCPTP increase the risk of rheumatoid arthritis due to Treg cell dysfunction (114). Therefore, using TCPTP agonists to overexpress or activate TCPTP seems to be a rational strategy against inflammatory bone resorption. This proposal can be supported by other studies. Zhang P. et al. found that TCPTP alleviated inflammatory responses and bone resorption in periodontal tissues *via* the JAK/STAT pathway in human oral keratinocytes and type 2 diabetes mellitus (T2DM) *db*/*db* mice (115). Zhang D.J. et al. demonstrated that TCPTP inhibited alveolar bone resorption in T2DM C57BL/6 wild-type mice *via* dephosphorylating CSF1 receptor (93). Consistently, our previous study series ascertained that TCPTP improves implant osseointegration in T2DM rats *via* ERK dephosphorylation (112). It is obvious that TCPTP could work as an effective potential target for preventing and treating inflammatory bone resorption. However, concerning the ubiquitous distribution and wide regulation of TCPTP related to glucose metabolism, immunoregulation, oncogenesis, and other various life processes in a cell or tissue-specific way, designing specific agents targeting TCPTP in specific cells, tissues or organs is a promising research direction and a novel aspect of osteoimmunology in the future.

## Conclusion

TCPTP bridges the two-way communication between immune cells and bone cells and it could work as a potential target for the prevention and treatment of inflammatory bone diseases, such as periodontitis, synovitis, and osteoarthritis. The proposal of identifying whether TCPTP can be used as an independent therapeutic target is feasible for the development of TCPTP agonists binding to a target of interest. This review provides clear insights into the potential roles of TCPTP in osteoimmunology, which may pave the way for further bone studies on osteoimmunological aspects.

## Author Contributions

Y-NW, SL, TJ, YF, and DZ made substantial contributions to conception, design, and acquisition of data. Y-NW, SL, WZ, and XX drafted the initial manuscript. XX and DZ critically reviewed it for important intellectual content. All authors contributed to the article and approved the submitted version.

## Funding

This work was supported by the National Natural Science Foundation of China (no. 82071148; no. 82001055), the Construction Engineering Special Fund of Taishan Scholars (TS201511106), the Key Project of Chinese National Programs for Research and Development (2016YFC1102705), the Shandong Provincial Natural Science Foundation (ZR2020QH158), the Fundamental Research Funds of Shandong University (2018GN024), the China Postdoctoral Science Foundation (2019M662371), and the Youth scientific research funds of School of Stomatology, Shandong University (2019QNJJ03).

## Conflict of Interest

The authors declare that the research was conducted in the absence of any commercial or financial relationships that could be construed as a potential conflict of interest.
